# Development of Target Sequence Capture and Estimation of Genomic Relatedness in a Mixed Oak Stand

**DOI:** 10.3389/fpls.2018.00996

**Published:** 2018-07-13

**Authors:** Isabelle Lesur, Hermine Alexandre, Christophe Boury, Emilie Chancerel, Christophe Plomion, Antoine Kremer

**Affiliations:** ^1^INRA, UMR 1202, Biodiversité Gènes et Communautés, Université Bordeaux, Pessac, France; ^2^HelixVenture, Mérignac, France

**Keywords:** oak, sequence capture, targeted sequence enrichment, genomic relatedness, NGS

## Abstract

Anticipating the evolutionary responses of long-lived organisms, such as trees, to environmental changes, requires the assessment of genetic variation of adaptive traits in natural populations. To this end, high-density markers are needed to calculate genomic relatedness between individuals allowing to estimate the genetic variance of traits in wild populations. We designed a targeted capture-based, next-generation sequencing assay based on the highly heterozygous pedunculate oak (*Quercus robur*) reference genome, for the sequencing of 3 Mb of genic and intergenic regions. Using a mixed stand of 293 *Q. robur* and *Q. petraea* genotypes we successfully captured over 97% of the target sequences, corresponding to 0.39% of the oak genome, with sufficient depth (97×) for the detection of about 190,000 SNPs evenly spread over the targeted regions. We validated the technique by evaluating its reproducibility, and comparing the genomic relatedness of trees with their known pedigree relationship. We explored the use of the technique on other related species and highlighted the advantages and limitations of this approach. We found that 92.07% of target sequences in *Q. suber* and 70.36% of sequences in *Fagus sylvatica* were captured. We used this SNP resource to estimate genetic relatedness in the mixed oak stand. Mean pairwise genetic relatedness was low within each species with a few values exceeding 0.25 (half-sibs) or 0.5 (full-sibs). Finally, we applied the technique to a long-standing issue in population genetics of trees regarding the relationship between inbreeding and components of fitness. We found very weak signals for inbreeding depression for reproductive success and no signal for growth within both species.

## Introduction

Predicting the evolutionary potential of natural populations is a major goal in many biological domains (e.g., evolutionary biology, landscape ecology, and conservation biology) given the global changes currently faced by organisms and populations. From an evolutionary perspective, the principal challenge is predicting the evolutionary changes required to track ongoing environmental changes and to identify key traits likely to respond to ongoing natural selection. These concerns are particularly important in the case of forest trees, which have long-generation times. Their evolutionary response must therefore occur within a very small number of generations. The prediction of evolutionary responses requires the estimation of essential genetic parameters, such as selection gradients, heritability, and evolvability, *in situ*, at the site at which selection is acting (Conner et al., 2003; Kruuk and Hill, 2008). Trait heritability can be estimated in situations in which the phenotypic similarity between individuals can be compared to their genetic similarity or relatedness (Ritland, 2000).

In animals, such as mammals and birds, such studies are generally performed on pedigreed populations (Kruuk, 2004). However, for trees, it is almost impossible to obtain pedigrees extending over more than two generations, at least over the lifetime of the scientist. Fortunately, recent developments in genomics, and the use of NGS sequencing have made it possible to measure the realized relatedness between individuals based on a large number of genetic markers, as it has been shown that the realized proportion of the genome identical by descent is more precisely estimated with a large number of molecular markers than with pedigree relationships (Kardos et al., 2015), these new methods thus open up new possibilities for the estimation of heritability and genetic variances *in situ* (Bérénos et al., 2014). Such approaches have already been implemented in trees (Castellanos et al., 2015). We addressed the aforementioned evolutionary questions, by identifying a large number of unlinked SNP markers in species of the Fagaceae family. These markers are widely distributed across the genome, encompassing genes and regions of biological interest, as well as regions assumed to be neutral.

Whole-genome shotgun sequencing is an easy way to sequence a genome randomly and to identify large numbers of molecular markers suitable for our objectives. However, shotgun sequencing may constrain marker development in highly repetitive genomes, such as that of oaks, which consists of 52% transposable elements (TEs), as reported by Plomion et al. (2018).

Targeted sequence capture coupled with NGS constitutes an efficient alternative approach to the exploration of genetic diversity in a very large number of genomic regions and specimens. The use of sequence capture techniques provides evolutionary biologists with easy access to nucleotide diversity, for addressing various research questions, as already demonstrated in arable crops (Zhou and Holliday, 2012), fruit (Tennessen et al., 2013), and forest trees (Neves et al., 2013; Holliday et al., 2016; Fahrenkrog et al., 2017).

Furthermore, these techniques provide high sequence coverage for a small set of target sequences, making it possible to multiplex several samples, thereby reducing the cost of large-scale applications, for population genetics studies, for example. Sequence capture techniques require access to a reference genome, but provide highly reproducible SNPs and markers with greater transferability across species than for other pangenomic marker systems (e.g., RADseq or GBS) (Harvey et al., 2016). Intra- and interspecific reproducibility is a prerequisite for comparative studies across populations or related species, even if sampling and molecular analysis are performed at different times. For example, George et al. (2011) developed a genomic capture approach in humans that successfully captured about 96% of coding sequences in monkeys (George et al., 2011). Similarly, in gymnosperms, a common capture design established for spruce and lodgepole pine (Suren et al., 2016) successfully captured more than 50% of the targeted bases with a coverage of at least 10×.

Our main objective here was to develop a large number of SNPs for estimating the genetic relatedness and inbreeding coefficient in a mixed oak stand containing two sister species: *Quercus petraea* and *Quercus robur*. We thus developed a targeted sequence enrichment strategy, explored its transferability to related species, and applied the detected markers to a long-standing question in tree population genetics: the relationship between inbreeding and fitness components.

## Materials and Methods

### Target Sequence Capture

#### Plant Material and DNA Extraction

**FIGURE 1:**
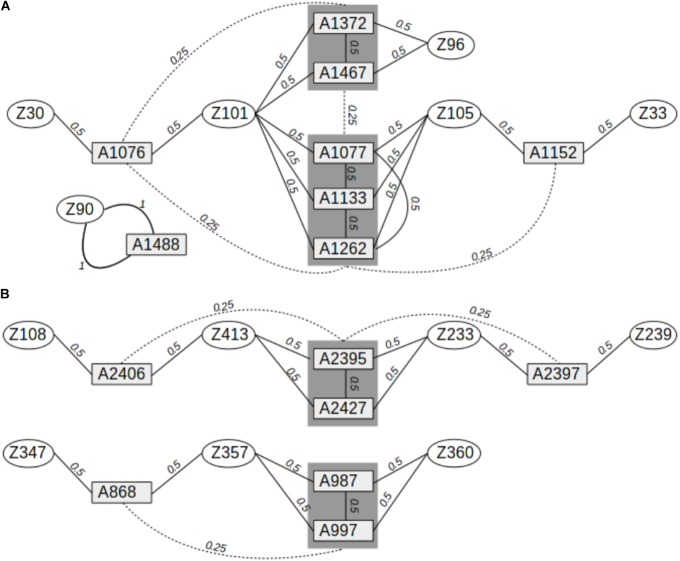
Pedigree relationships between *Q. robur* and *Q. petraea* siblings in the sequence capture experiment. **(A)** Pedigree relationships between 8 *Q. petraea* siblings and their parents. **(B)** Pedigree relationships between 7 *Q. robur* siblings and their parents. Rectangles and ellipses correspond to siblings and parents, respectively. Numbers connecting trees correspond to expected genetic relatedness between individuals, based on their known pedigree.

Leaves were collected from 278 adult oak trees and 15 siblings (8 *Q. petraea* and 7 *Q. robur*) in a mixed oak stand (*Q. petraea–Q. robur*) located in the Petite Charnie State Forest in Western France (latitude: 48.086°N; longitude: 0.168°W). This population corresponds to cohort #1b described by Truffaut et al. (2017). The trees were all cut between 1989 and 1993, but were grafted and maintained in a common garden in a nursery located in Guéméné (latitude: 47.63°N; longitude: −1.89°W). Leaves were sampled from these grafted plants for DNA extraction. The 15 siblings were sampled during the natural regeneration of the adult trees in the Petite Charnie Forest and are part of cohort #2 described by Truffaut et al. (2017). The parents of the siblings were identified by molecular parentage analysis in a previous study (Truffaut et al., 2017), and pedigree relationships were inferred between the parents and their offspring, and between the offspring (**Figure 1**).

We also collected leaves from two adult beech trees (*Fagus sylvatica*) from St. Symphorien, on a tributary of the Ciron river, in south-west France (latitude: 44.25°N; longitude: 0.29°W) and two adult cork oak trees (*Quercus suber)* growing at the INRA Research Station at Pierroton in south-west France (latitude: 44.44°N; longitude: 0.46°W). We considered a total of 300 samples in all, as three adult trees from the Petite Charnie forest were sampled twice.

For DNA extraction, leaves were frozen and stored at −80°C. DNA was extracted with the QIAGEN DNeasy Plant Maxi Kit and DNA quality and quantity were assessed with a spectrophotometer (NanoDrop Thermo Fisher Scientific, Waltham, MA, United States) and a fluorometer (Tecan Infinite F200, Männedorf, Switzerland) with a Broad Range Quant-iT dsDNA kit (Thermo Fisher Scientific, Waltham, MA, United States). For each replicated individual, DNA was extracted independently from each of the two samples, independent libraries were constructed, and replicates were sequenced in separate proton sequencing runs.

#### Target Sequence Selection and Probe Design

We used in-solution hybridization-based sequence capture technology, based on the results of Mamanova et al. (2010). These authors compared the performance of several target-enrichment techniques, assessed on the basis of several criteria: percentage of target sequences captured, proportion of sequencing reads on target, variability of sequencing coverage across target regions, reproducibility, cost, ease of use, and minimum amount of DNA required. Given the number of samples studied, the target size imposed by our resources (2.9 Mb) and the relatively large proportion of repetitive sequences, hybridization-based sequence capture appeared to be the most relevant method in our case.

The haploid version of the *Q. robur* genome (haplome V2.3), available from: http://www.oakgenome.fr/ and described by Plomion et al. (2018), was used for probe design. The oak genome consists of 25,808 predicted protein-coding genes spread over 1,409 scaffolds. The oak genome is highly repetitive. We therefore limited the length of target sequences to 150 bp, when necessary, to avoid repetitive sequences. Target sequences were selected on the basis of previous results for genetic diversity and the expression of genes of ecological and physiological relevance. Indeed, over the past 10 years, various genetic surveys have been conducted to identify expressed candidate genes, outlier genes displaying species or population genetic differentiation, or genes displaying significant genotype–phenotype or genotype–environment associations. We reviewed all these surveys and used relaxed thresholds of selection to identify candidate sequences for genomic capture (**Table 1**). As our resources were limited to a total sequence length of 2.9 Mb for capture, we could not consider entire genes as targets for probe design. We therefore selected target sequences within each gene, depending on its length. For genes of less than 1.5 kb in length, we identified a single 150 bp target sequence located in an exon. Longer genes were artificially subdivided into three regions, and we selected two 150 bp target sequences located in two extreme regions of the gene: one within an exon, and the other within an intron–exon transition (Supplementary File 1). In total, our capture experiment included 9,748 candidate genes. We completed the selection and design of target sequences for genomic capture, by including 150 bp sequences located in intergenic regions. These sequences were selected with a 100 kb sliding window. We examined 8,936 windows, and retained a 150 bp sequence at the beginning of the window only if no other target sequence had previously been identified in the window (**Table 1**). If the target sequence colocalized with a TE, it was shifted to 150 bp further along in the genome.

**Table 1:** List of candidate target sequences selected before probe construction.

Set of sequences	Selection criteria of target sequences	Phenotypic or environmental variation	Number of candidate target sequences	Reference
1	Species divergence	Unknown	17	Guichoux et al., 2013
2	Species divergence	Unknown	1,560	Leroy et al., 2018
3	Genotype–phenotype association	Time of leaf unfolding	681	Unpublished
4	Genotype–phenotype association	Time of leaf unfolding	40	Alberto et al., 2013
5	Genotype–environment association	Temperature	740	Unpublished
6	Differential expression	Response to waterlogging	4,694	Le Provost et al., 2016
7	Differential expression	Dormancy	6,069	Ueno et al., 2013
8	None (intergenic regions)	Unknown	1,822	This study
Total			15,623	

*Eight sets of sequences were selected in total (see section “Materials and Methods”): five were reported in earlier studies (Alberto et al., 2013; Guichoux et al., 2013; Ueno et al., 2013; Le Provost et al., 2016), two are unpublished, and the last one is provided here.*

Once target sequences had been identified, we retained only those with a GC content between 30 and 60%, as suggested by Chilamakuri et al. (2014). We avoided repetitive regions of the genome by aligning candidate target sequences against the oak genome with BLAT v. 35x1, using default parameters (Kent, 2002), and we retained target sequences with fewer than 10 alignments on the oak genome that were distant from TEs.

Following this strategy, we identified 15,623 candidate target sequences, which were sent to Agilent Technologies (Agilent Technologies, Santa Clara, CA, United States) for the design of 120 bp probes.

#### Library Preparation

Our targeted enrichment procedure was based on Agilent’s SureSelect target enrichment system for Ion Torrent Proton sequencing (Thermo Fisher Scientific, Waltham, MA, United States). We randomly assigned the 300 DNA samples to 20 groups, each corresponding to a proton sequencing run. The 15 samples in each run were labeled (indexed 1–15). We assessed reproducibility, by duplicating three samples corresponding to three individuals. For the three duplicated samples, DNA was extracted separately from the two samples, independent libraries were constructed and sequencing was performed in separate runs. A pre-capture library was prepared for each sample, using the NEBNext^®^ Fast DNA Library Prep Set for Ion Torrent^TM^ from New England Biolabs (Ipswich, MA, United States) according to the manufacturer’s instructions: 400 ng of genomic DNA was sheared, with an M220 focused ultrasonicator (Covaris, Inc., Woburn, MA, United States), to yield 200 bp fragments. Each sheared DNA sample was subjected to end repair and ligated to barcoded adapters. We then selected DNA fragments of 300 bp in size by two consecutive Agencourt^®^ AMPure^®^ XP steps (Beckman Coulter, Inc., Brea, CA, United States): 0.7× then 0.15×. The libraries were subjected to 11 cycles of amplification. Each library was quantified with a Qubit Fluorometer, with the Qubit^TM^ dsDNA HS Assay Kit (Thermo Fisher Scientific, Waltham, MA, United States). Then equimolar pools of three libraries were prepared (250 ng for each library) for target enrichment.

#### Target Enrichment

The size of the DNA library was limited by the use of in-solution capture, which requires an excess of probe over template. Hybridization to the probes was carried out for 24 h at 65°C, according to the Agilent protocol, in a thermocycler, with 750 ng of library. Following the hybridization and washing steps, the recovered targeted DNA fragments were amplified in KAPA HiFi HotStart ReadyMix (Kapa Biosystems, Wilmington, MA, United States) for 40 s at 98°C, followed by eight cycles of 30 s at 98°C, 30 s at 62°C, and 30 s at 72°C, with a final extension for 5 min at 72°C. The captured library pools were quantified by qPCR on a LightCycler^®^ 480 System (Roche Molecular Diagnostics), with the Ion Library TaqMan^TM^ Quantification kit. In total, 20 pools of 15 libraries were used in equimolar amounts, with a final concentration of the pooled samples of 5 pM for sequencing on an Ion Proton System (Thermo Fisher Scientific, Waltham, MA, United States).

#### Sequence Enrichment

For each sample, high-quality Ion Torrent proton reads were demultiplexed and subjected to quality control with Torrent suite V5.0.5 (Thermo Fisher Scientific). Reads were independently aligned with the oak genome, using the Torrent Mapping Alignment Program (TMAP, Thermo Fisher Scientific) and the default parameters for the Torrent suite. We estimated target enrichment by quantifying the proportion of sequencing reads correctly aligned with the target sequences. For each sample, this “on-target” set of reads was considered for further analysis. We investigated the coverage of target sequences and calculated the percentage of the length of the target covered by at least one read. These analyses were performed with custom scripts developed in Python V2.7.2.

### SNP Detection and Population Genetics Analyses

#### SNP Detection and Filtering

For each sample (including cork oak and beech, which were used to test the transferability of the capture probes to related species), SNPs were independently called, first with the *mpileup* function of samtools V1.3.1, and then with the *bcftools* function V1.1-60-g3d5d3d9 (Li et al., 2009). We considered only diallelic variants with a coverage of more than 10×. The minimum allele frequency (MAF, upper case used at the individual level) within an individual, calculated on the basis of all the reads containing the SNP, was set to 30%. A nucleotide polymorphism was considered to be an SNP, if at least one individual was found to be heterozygous at the position concerned within the whole population of 300 samples. For studies of relatedness between individuals, we considered only the 293 oak trees from the Petite Charnie forest (278 adults + 15 siblings). The SNP detection pipeline is described in Supplementary File 2. We performed multiple controls and filtering steps in the Petite Charnie population (i.e., 293 trees). We removed all trees for which more than 20% of the SNPs were missing. Similarly, SNPs scored in less than 95% of the trees were removed from the dataset, together with SNPs located on the 538 unanchored scaffolds of the oak genome (Plomion et al., 2018).

#### Assignment of Individuals to Species

For the assignment of each individual to a species, we retained markers in Hardy–Weinberg equilibrium located at least 1,000 bp apart, to avoid a redundancy of marker information due to linkage disequilibrium. We assigned each individual to a species (i.e., cluster) with the fastSTRUCTURE V1.0 algorithm (Raj et al., 2014). We allowed one to five clusters, with default parameters, and the DISTRUCT algorithm was run over assignments based on cluster numbers of two to five, to determine the most likely number of clusters. We assigned individuals strictly to one species (*Q. robur* or *Q. petraea*) excluding admixed individuals on the basis of the posterior probability of each individual belonging to one of the clusters.

#### Estimation of Genomic Relatedness and Inbreeding

We investigated the genetic relatedness between trees, by removing markers in linkage disequilibrium (*r*^2^ > 0.4) with their neighbors, using the indep-pairphase function of PLINK v1.90b3.34 (Purcell et al., 2007) (window size of 50 markers). We performed a Hardy–Weinberg equilibrium exact test (Wigginton et al., 2005) with the ±hardy function of PLINK, and *P*-values were adjusted according to the FDR method of Benjamini and Hochberg (1995), with the *R* function p.adjust (Benjamini and Hochberg, 1995). Only markers with a *P*-value greater than 0.05 after correction were retained. From these markers, we computed the Fst for each marker common to both populations (*Q. petraea* and *Q. robur*) with the function Fst from the R package pegas (Paradis, 2010). Finally, we considered six sets of markers defined on the basis of MAF, considered here at the population level (maf, in lower case, for population level): we selected markers with a maf exceeding a threshold of 0.01, 0.05, 0.1, 0.15, 0.3, or 0.4 (Supplementary File 2).

For each species and each set of SNPs, the genomic relatedness matrix (*G*) between individuals was estimated as:

$$\text{G}=\frac{(\text{M}-\text{P})\cdot (\text{M}-\text{P}{)}^{\prime }}{2\sum {\text{p}}_{\text{i}}\cdot (1-{\text{p}}_{\text{i}})}$$

where *M* is an *n*^∗^*m* matrix of genotypes scored as −1, 0, or 1 for homozygote, heterozygote, alternative homozygote, *P* is a *n*^∗^*m* matrix of allele frequencies computed as 2 (*p_i_* ± 0.5), *p_i_* is the maf at locus i, *n* is the number of individuals, and *m* is the number of markers, as described by VanRaden (2008), with the kin function of the R package synbreed (VanRaden, 2008; Wimmer et al., 2012).

As indicated above, 15 offspring from the Petite Charnie stand were previously genotyped for 82 SNPs, and their parents were identified by parentage analysis (Truffaut et al., 2017). The 15 siblings were either full-sibs or half-sibs from 13 different adult trees, resulting in a total of 54 pairwise-related individuals. Eight siblings were the offspring of six adult *Q. petraea* trees, whereas seven were the offspring of seven adult *Q. robur* trees. Four different pedigree relationships were identified among these 54 pairs of trees: parent–offspring selfed, parent–offspring, full-sib–full-sib, and half-sib–half-sib. These relationships corresponded to three different expected coefficients of relationship: 1, 0.5, and 0.25 (**Figure 1**). For the 54 pairs of trees, we compared genomic relatedness (*G*) with the expected pedigree relatedness. Finally, we also calculated the genomic relatedness based on the 82 SNPs obtained in a previous study (Truffaut et al., 2017). In the genomic relatedness matrix (*G*), diagonal elements (*G*_ii_) correspond to the relatedness of each individual i to itself relative to population allelic frequencies. In a theoretical population, at equilibrium, with no inbreeding, each individual should have a *G*_ii_ of 1. Inbreeding is thus assessed as *G*_ii_–1 (VanRaden, 2008). The deviation from 0 is interpreted as the individual level of inbreeding relative to the population: the coefficient of genomic inbreeding can be positive (i.e., individuals are more homozygous than expected from population allelic frequencies) or negative (i.e., individuals are less homozygous than expected from population allelic frequencies).

#### Correlation Between Genomic Inbreeding and Fitness

We used two traits as proxies for fitness: (i) the reproductive success of each adult tree, as assessed by the parentage analysis of 2,500 offspring and the adult trees, and (ii) the growth of each tree, as assessed by measuring stem circumference at breast height when the trees were cut. The method used to assess reproductive success has been described elsewhere (Truffaut et al., 2017). For each species we used the glm function of R to generate a generalized linear model with the number of offspring regressed against environmental variables and the inbreeding level, according to the formula:

$$g(F_{\text{i}})=\alpha +\beta_{1}X_{\text{i}1}+\gamma I_{\text{i}}+\varepsilon_{\text{i}}$$

where *F*_i_ is the reproductive success of individual *i*, α is the intercept, β_1_ is the regression coefficient associated with the first axis of principal component analysis (PCA) on the five environ is the first PC value extracted from this PCA, *I_i_* is the inbreeding coefficient of individual *i* associated with the regression coefficient γ, 𝜀*_i_* is the residual error, and *g* is a log-link function associated with the Poisson distribution data. Independent variables were centered such that the intercept of the model corresponded to the phenotypic mean for the population. This transformation had no effect on the regression coefficient values, their standard error or the associated *P*-values. We applied a similar approach to the circumference, except that we used a linear model, as circumference is a normally distributed quantitative variable, and we added the age at which each tree was cut as an independent variable (range: 78–102 years).

## Results

### Target Sequence Capture

#### Agilent Probe Design

The 3P *Q. robur* reference genome was used for probe design (Plomion et al., 2018).

The mean size of the target sequences was 150 bp and the probes were 120 bp long. One or two non-overlapping probes were therefore designed per target sequence, resulting in a total of 33,931 120 bp probes designed with SureSelect eArray software (Agilent Technologies, Santa Clara, CA, United States). These probes covered a total of 2,897,647 bp (i.e., 0.39% of the estimated haploid genome size). In total, 23,704 probes targeted 11,446 (44.35%) of the 25,808 predicted protein-coding genes and 10,227 probes targeted intergenic regions (**Table 2**). In total, 11,120 probes (46.91%) targeted exons, whereas 6,731 (28.40%) targeted intronic regions and 5,853 (24.69%) targeted exon–intron regions.

**Table 2:** Number of probes and target sequences in intergenic and genic regions.

Sequence type	Number of probes	Number of targets
Intergenic region	10,227	4,031
Genic region	23,704	11,446
Exon	11,120	4,960
Intron	6,731	2,991
Intron–exon junction	5,853	3,495
Total	33,931	15,477

*A total of 33,931 120 bp probes were designed to capture 15,477 target sequences.*

The probes designed successfully avoided repeated regions within the genome, as fewer than 10 alignments with the oak genome were identified for 97.36% of the probes (33,034 probes).

#### Target Sequences Identification

We selected a total of 15,623 genomic regions for capture (i.e., 2,914,160 bp), as described in **Table 1**. Agilent Technologies successfully designed 33,931 probes for 15,477 target sequences (99.07%). Among the target sequences, 4,031 (26.05%) corresponded to intergenic regions and 11,446 (73.95%) corresponded to genes (**Table 2**). In total, 4,960 (43.33%) sequences corresponded to exons, whereas 2,991 (26.13%) sequences were located in intronic regions, and 3,495 (30.54%) were located in exon–intron regions. The 4,031 intergenic target sequences were distributed as follows: an initial set of 1,796 intergenic target sequences (**Table 1**), with 2,235 sequences of 150 bp in length used as putative selectively neutral control regions for population genetic analyses. These control regions were evenly distributed over the genome.

#### Efficiency of Target Enrichment

The probes designed captured 15,477 target sequences, corresponding to 2,897,647 bp of *Q. robur* DNA. In total, 20 pools of 15 individuals each were independently sequenced with the Ion Torrent Proton sequencing system (i.e., 300 samples), with three samples sequenced twice. Each sequencing run produced between 65,426,948 and 134,977,869 reads (Supplementary File 3). Target enrichment was assessed by aligning the reads with the oak genome: on average, for each run, 25.20% of the reads captured 97.19% (i.e., 15,042) of the target sequences (Supplementary File 3). On average, 95.47% of the length of the target sequences was captured, and the mean coverage depth over all samples was 96.81×, (range: 48.39 to 161.67×). Coverage length was 95.82% and 98.24× coverage was achieved for the set of *Q. robur* and *Q. petraea* samples from the Petite Charnie stand (i.e., 296 samples). The size of the sequenced reads ranged from 140 to 190 bp (mean: 174 bp). The length of the sequencing reads was significantly positively correlated with the percentage of on-target sequences (adjusted *R*^2^= 0.2892, *P*-value = 2.2*e*–16) (Supplementary File 4).

#### SNP Calling

**FIGURE 2:**
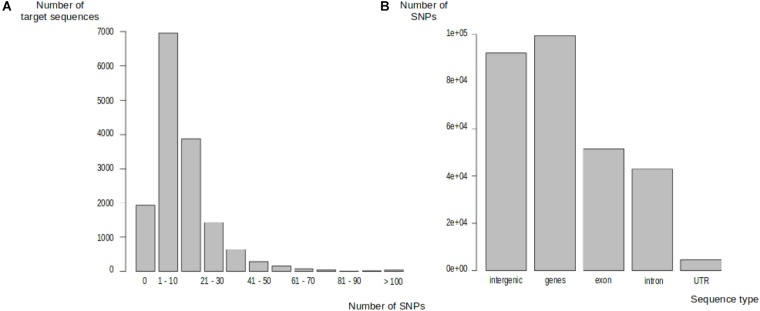
Distribution of SNPs in target sequences. **(A)** Distribution of SNPs in target sequences. **(B)** Distribution of SNPs in target sequences based on sequence types.

We identified 191,281 polymorphic sites in one of the 297 trees, distributed between 13,572 target sequences (87.69%). The number of SNPs in target sequences ranged from 1 to 603 (**Figure 2A**). Most of the target sequences displaying polymorphism (10,419, 67.32%) contained between 1 and 20 SNPs. The SNPs were, thus, evenly spread over most of the target sequences. We classified these SNPs into genic and intergenic sites on the basis of the oak gene model (Plomion et al., 2018). There were 191,281 SNPs in total: 92,002 (48.10%) were located in intergenic regions and 99,279 (51.90%) were located within genes. In total, 51,536 SNPs (51.91%) were exonic, 43,075 SNPs (43.39%) were intronic, and 4,668 SNPs (4.70%) were located in UTR regions (2,131 in the 5′UTR and 2,537 in the 3′UTR) (**Figure 2B**). On average, 7.28 and 10.49 SNPs were detected every 100 bp in genic and intergenic regions, respectively. Intergenic regions were much less covered than genic regions, with a median sequencing depth of 97 and 63 in genic and intergenic regions, respectively. Finally, we detected a mean of 13,219 SNPs per tree within the Petite Charnie population.

#### Reproducibility

**FIGURE 3:**
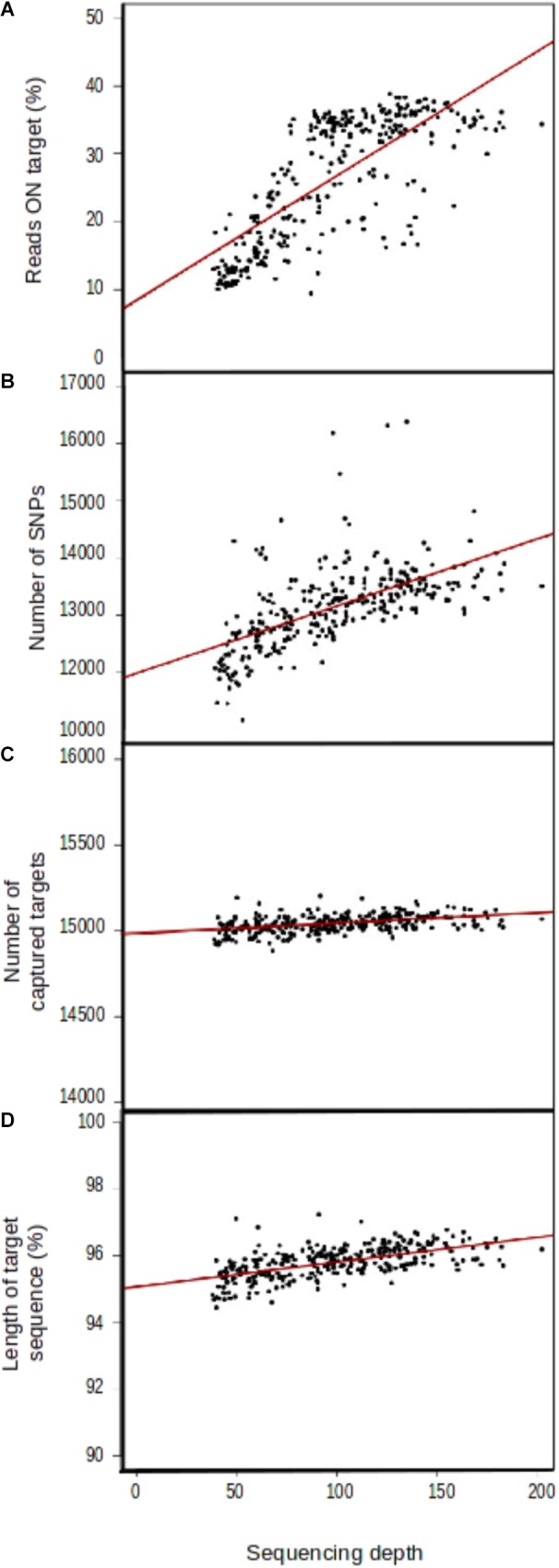
Correlation between sequencing depth and genomic capture efficiency parameters. **(A)** Mean number of reads aligned with target sequences, **(B)** mean number of SNPs per sample, **(C)** number of captured target sequences, and **(D)** target sequence length.

Mean sequencing depth differed considerably between proton sequencing runs (Supplementary File 5), even though number of samples per pool in the sequencing runs was identical (15). Four variables were correlated, to some extent, with sequencing depth: the percentage of reads on target (*r*^2^ = 0.182753, **Figure 3A**), the number of SNPs detected (*r*^2^ = 1.165*e*+01, **Figure 3B**), the number of captured target sequences (*r*^2^ = 6.175*e*–01, **Figure 3C**), and the mean length of the captured sequences (*r*^2^ = 7.401*e*–03, **Figure 3D**).

The genomic capture assay was repeated twice for three oak genotypes of the Petite Charnie population. For each genotype, the number of captured targets and the length of the capture sequence were similar (**Table 3**). Given the different sequencing depths of the different runs and the stringent filters applied for SNP detection (intra-individual MAF = 30%, depth ≥ 10), for each individual, we did not capture the entire set of targeted SNPs (**Table 3**) (80.31% for tree #049, 72.32% for tree #288, and 60.90% for tree #402). Nevertheless, when captured in both replicates, the same alleles were almost systematically correctly retrieved (**Table 3**) (99.9% similarity). When considering all sites (polymorphic sites and monomorphic sites covered by at least by 20×), the percentage of genotype similarity among replicates was 99.86, 99.65, and 99.27% for tree #049, tree #288, and tree #402, respectively. As expected, decreasing the intra-individual MAF for SNP detection from 30 to 10% increased the number of SNPs detected. This also made it possible to increase the proportion of targeted SNPs detected for all samples (80–84% for tree #049, 60–68% for tree #402, and 72–78% for tree #288). Again, when variants were detected in both replicates, allele similarity was maintained (99.9%). For all samples, sequencing depth exceeded 10× for most of the SNPs detected in only one of the two replicates (**Table 4**). We conclude that the individuals were monomorphic at these loci. However, increasing the sequencing depth threshold from 10 to 20× should significantly increase the number of SNPs detected in both replicates.

**Table 3:** Statistics of the replicated samples.

Run ID	Tree ID	Number of captured target	Captured length (%)	Depth (*X*)	SNPs	Common SNPs	Identical alleles	Different alleles
G	049	15,030 (97.11%)	95.78	137	13,804	12,422	12,417	5
R	049	15,120 (97.69%)	96.52	179	14,080			
I	402	15,053 (97.26%)	95.62	47	14,291	10,843	10,832	11
R	402	15,069 (97.36%)	96.23	124	16,318			
J	288	14,884 (96.17%)	94.62	67	12,431	10,908	10,900	8
R	288	15,038 (97.16%)	95.90	158	13,561			

*Comparison of the number of SNPs detected for the three replicated samples.*

**Table 4:** Statistics of the replicated samples.

Tree ID	Number of polymorphic sites	Repeat 1			Repeat 2		
		Htz	Hmz	NA	Htz	Hmz	NA
049	15,462	13,804	1,658	0	14,080	1,382	0
402	17,805	12,330	4,765	710	16,318	1,486	1
288	15,084	12,431	1,951	702	13,561	1,520	3

*For each genotype (tree #049, tree #402, and tree #288), the number of polymorphic and monomorphic sites detected for each replicate (rep #1 and rep #2) was compared with the total number of sites found to be polymorphic in at least one replicate. Htz is the number of polymorphic sites; Hmz is the number of monomorphic sites; and NA is the number of sites for which sequencing depth was insufficient for the detection of polymorphism.*

Finally, we were also able to test for SNP reproducibility, as 25 SNPs identified by SNP calling were included in an earlier SNP scoring method used in a previous study of the same trees (Truffaut et al., 2017). Indeed, the 278 adult oak trees of La Petite Charnie had already been scored for 82 SNPs for a parentage analysis, with a MassARRAY^®^ System 16 and iPLEX^®^ 17 chemistry (Agena Bioscience, San Diego, CA, United States) and 25 of these SNPs were also used in this study. The SNPs identified by the two methods were similar for the two methods except for two trees, for which differences were observed at multiple SNPs. We suspect that these differences result from labeling errors, given that the two analyses were conducted 3 years apart, with different DNA extracts. These two trees were therefore removed from subsequent analyses. A total of 25 SNPs and 250 individuals was scored with both methods (sequence capture and sequenom) giving two sets of 6,250 genotypes. We thus compared the two sets, and over the 6,250 repeated genotypes, 97.67% was concordant (i.e., similar) between the two methods.

#### Transferability

We studied the transferability of the targeted sequence capture technology to other species, by including two cork oak (*Q. suber)* and two beech (*F. sylvatica)* samples in our study. An alignment of cork oak reads against the 3P oak genome showed a significant level of target enrichment: on average, for both samples, 15.86% of the reads captured 92.07% (i.e., 14,283 and 14,217) of the target sequences (**Table 5**). When captured, target sequences were covered over 87.18% of their length on average, and the mean depth of coverage over the two samples was 56.03×. Lower values were obtained for the two beech specimens. On average, 8.93% of the reads captured 70.63% (i.e., 10,851 and 11,014) of the targeted sequences. Length coverage was only 51.60%, and sequencing coverage was significantly lower, at 26.30×.

**Table 5:** Interspecific transferability statistics.

Species	Reads ON target (%)	Captured sequences (%)	Captured length (%)	Sequencing depth (*X*)	Number of SNPs
*Q. petraea*, *Q. robur*	25.20	97.19	95.82	98.24	13,219
*Q. suber*	15.86	92.07	87.18	56.03	9,093
*F. sylvatica*	8.93	70.63	51.60	26.30	3,000

*For each species, the values for the percentage of reads on target per tree, the percentage of captured sequences per tree, the percentage length in captured sequences per tree, the sequencing depth per tree, and the number of SNPs per tree are provided. For Q. petraea and Q. robur, 293 trees (adults and siblings) from the mixed oak stand located in the Petite Charnie State Forest were considered. Q. suber data were obtained from two adult trees located at the INRA Research Station at Pierroton. F. sylvatica data were obtained from two adult trees located in St. Symphorien. In total, 15,477 target sequences selected in the 3P Q. robur genome were considered for all species.*

When considering *Q. robur* and *Q. petraea* trees only (i.e., 293 trees), we identified 13,219 SNPs per sample, on average (**Table 5**). Smaller numbers of SNPs were detected in the other two species: 9,093 and 3,000 SNPs in cork oak and beech, respectively.

**FIGURE 4:**
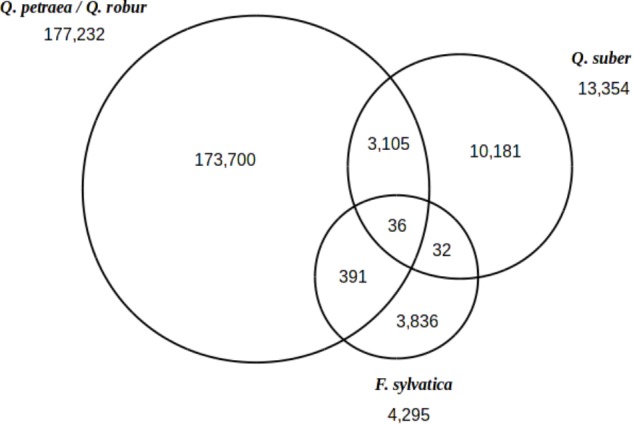
Inter-specific transferability of SNPs. Venn diagram showing the distribution of 191,281 polymorphic sites between *Q. petraea/Q. robur*, *Q. suber*, and *F. sylvatica*.

When considering all 297 trees studied here (including 2 *Q. suber* and 2 *F. sylvatica* genotypes), we identified a total of 191,281 polymorphic sites heterozygous in at least one of these trees (**Figure 4**). In total, 177,232 polymorphic sites were identified in *Q. robur* and *Q. petraea*, and 13,354 and 4,295 sites were identified in *Q. suber* and *F. sylvatica*, respectively. A set of 36 SNPs was found to be common to all three species, as 10,181 SNPs were specific to *Q. suber* (i.e., 76% of the *Q. suber* SNPs) and 3,836 SNPs were specific to *F. sylvatica* (i.e., 89.31% of the *F. sylvatica* SNPs). As expected, more SNPs were shared between the *Quercus* sp. than between *Quercus* and *Fagus*.

### Population Genetics in the Petite Charnie Forest Stand

#### Species Assignment and Interspecific Differentiation

**FIGURE 5:**
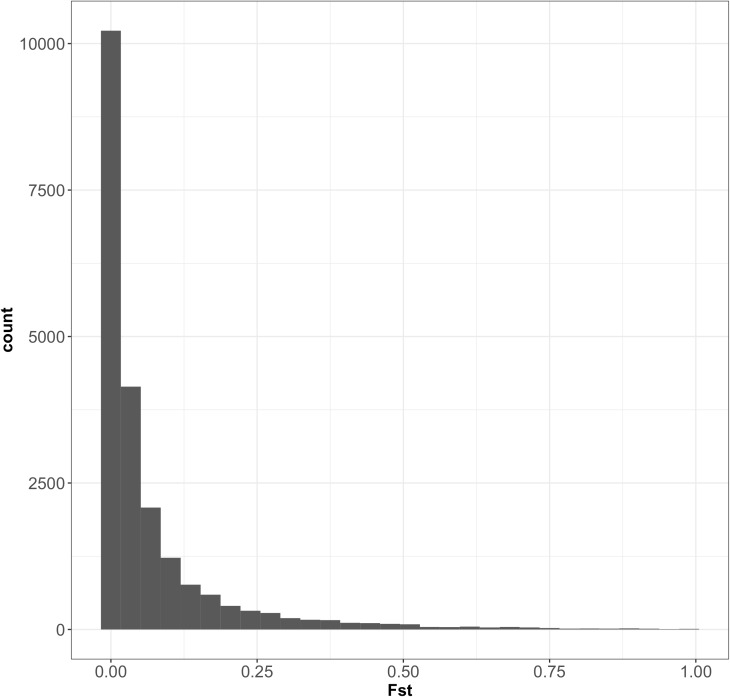
Distribution of Fst between *Q. petraea* and *Q. robur* over 21,331 markers. The 21,331 SNPs correspond to a set of markers common to *Q. petraea* and *Q. robur* (also considering those fixed within populations).

According to fastSTRUCTURE, the most probable number of clusters was 2, consistent with the findings of a previous analysis performed on oak trees in the same forest, with 82 SNPs (Truffaut et al., 2017). Individual trees were assigned to the two species according to the value of the admixture coefficient (*q*) obtained with fastSTRUCTURE software. Trees were assigned to three groups on the basis of threshold values of *q*: *Q. petraea* purebreds (*q* ≥ 0.9), admixed trees (*q* 0.1–0.9), and *Q. robur* purebreds (*q* ≤ 0.1), as described in Truffaut et al. (2017). The results of the fastSTRUCTURE assignment were similar to that of the published results obtained with STRUCTURE (Truffaut et al., 2017), except for two individuals assigned to *Q. robur* by Truffaut et al. but considered admixed in our study. These two trees had admixture values very close to the *q* threshold values in study of Truffaut et al. (2017). Population maf values and heterozygosity distribution within species are presented in Supplementary File 6. Of the 45,429 SNPs detected in *Q. petraea* and the 51,886 SNPs detected in *Q. robur*, 21,331 were common to these two species. *F*st values for all the 21,331 markers common to *Q. petraea* and *Q. robur* showed an L-curve distribution, with a large number of SNPs displaying very low levels of interspecific differentiation (**Figure 5**). The mean and median *F*st values between the two species were 0.069 and 0.019, respectively, suggesting that these two species display no clear differentiation over a large part of their genome.

#### SNP Detection and Filtering

Successive filtering steps on the 191,281 polymorphic sites resulted in various numbers of markers. The final filtering step based on population maf resulted in the lowest number of markers for maf = 0.4 and the highest for maf = 0.01, with 1,561–33,131 usable markers for *Q. robur* and 1,454–32,047 for *Q. petraea*, respectively (see Supplementary File 2 for details).

#### Genomic Relatedness

**FIGURE 6:**
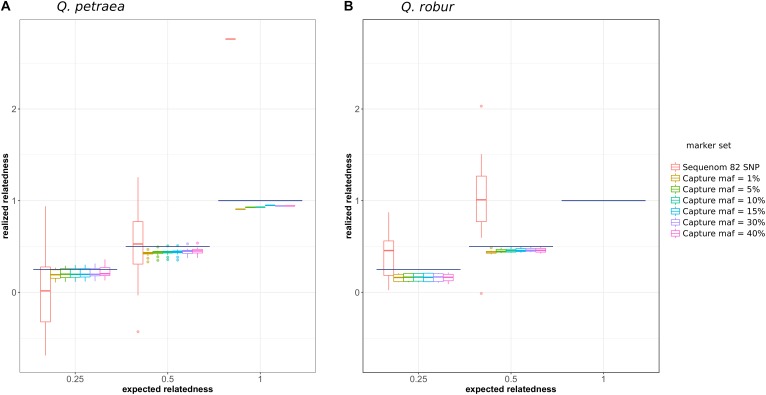
Comparison of expected (pedigree-based) and realized genomic relatedness for different marker sets (**A** for *Q. petraea* and **B** for *Q. robur*). Expected relatedness based on pedigree relationship is illustrated in **Figure 1**, and shown on this graph by bold horizontal lines [for *Q. petraea* **(A)** and *Q. robur* **(B)**]. Colored box plots correspond to realized genomic relatedness, as determined with different subsets of SNPs screened according to different thresholds of minimum allele frequency (maf). The pink large-range box plots corresponds to the realized genomic relatedness obtained with the 82 SNPs in the sequenom assay (see text). The number of pairwise relatedness estimates for each expected relatedness category are as follows: *Q. petraea* *n*_0.25_ = 14, *n*_0.5_ = 18, *n*_1_ = 1; *Q. robur* *n*_0.25_ = 6, *n*_0.5_ = 16, *n*_1_ = 0. The expected relatedness coefficients are extracted from **Figure 1**.

**FIGURE 7:**
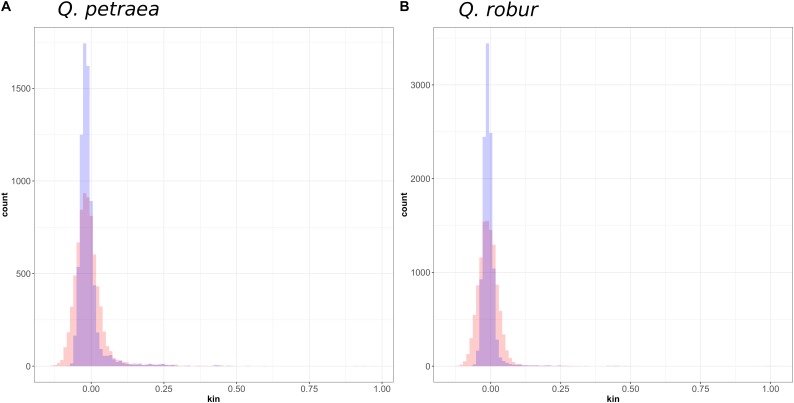
Distribution of genomic relatedness between **(A)** *Q. petraea* and **(B)** *Q. robur* trees of the Petite Charnie forest computed with marker sets selected at MAF threshold of 1% (blue) and 40% (red).

We first compared the expected relationship coefficient derived from pedigree relationships and realized genomic relatedness in the two parent–offspring groups of known pedigree relationships, for 54 individual pairs (**Figure 1**). Considering only genomic relatedness estimated by genomic capture, very minor differences in mean values were observed for numbers of markers between 32,500 markers (maf = 0.01) and 1,500 markers (maf = 0.4). However, this difference in the number of markers had a slight impact on precision, as the variance of the estimate was lower for larger numbers of markers (**Figure 6**), a finding supported by the overall distribution of relatedness between individuals (**Figure 7**). Thus, the use of numerous rare alleles has no major effect on the prediction of genomic relatedness. Realized genomic relatedness was slightly lower than expected, in both species (**Figure 6**). Conversely, when estimated with 82 SNPs only, genomic relatedness was scattered around the expected value (**Figure 6**). The fact of including non-neutral markers (located in exons) in the SNPs sets had no impact on the genomic relatedness estimation (data not shown here).

At population level, relatedness coefficients were distributed around a mean value of 0 (**Figure 7**), as expected, given the method used to calculate relatedness. However, we can consider overall mean genetic relatedness to be low within natural populations of *Q. petraea* and *Q. robur.* Among parents (i.e., excluding the 15 offspring) with a population maf = 0.05, only 20 (*Q. robur*) and 40 (*Q. petraea*) pairs of trees had a genomic relatedness of more than 0.25 (expected for first-cousin relationship or half-sibs) and only three (*Q. robur*) and two (*Q. petraea*) pairs had a genomic relatedness of more than 0.5 (expected for full-sibs), among 8,151 (*Q. robur*) and 10,150 (*Q. petraea*) pairwise estimates.

#### Correlation Between Inbreeding and Fitness-Related Traits

**FIGURE 8:**
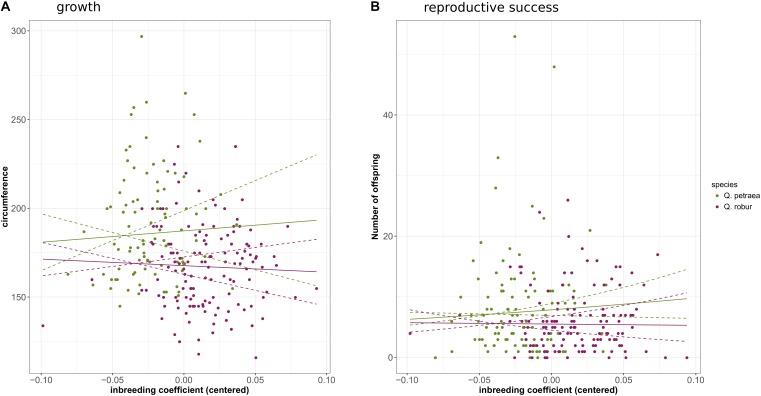
Correlation between genomic inbreeding and growth **(A)** or reproductive success **(B)**. The solid curves correspond to the regression of growth or reproductive success against inbreeding coefficient according to the estimated regression coefficients. The doted lines correspond to the 95% confidence interval of the regressions.

Genomic inbreeding coefficients were estimated separately for each species from the *G* matrix calculated with the 1% population maf threshold and markers common to the two species. Overall rates of inbreeding within the two oak species were low (Supplementary File 7). However, one *Q. petraea* tree had a very high inbreeding value (0.58), and was discarded from the analysis. Overall, the individuals of *Q. robur* were more inbred (mean inbreeding = 0.068, *SD* = 0.030) than the individuals of *Q. petraea* (mean inbreeding = 0.037, *SD* = 0.056). GLM analysis showed the number of offspring to be significantly negatively correlated with inbreeding level in *Q. petraea* (coefficient = −3.62, *P*-value = 6.06*e*-3), whereas this relationship was not significant in *Q. robur* (coefficient = −1.81, *P*-value = 0.114) (**Figure 8B**). There was no significant correlation between genomic inbreeding and circumference at breast height [*Q. petraea*: coefficient = −25.39, *P*-value = 0.83; *Q. robur*: coefficient = −36.14, *P*-value = 0.58 (**Figure 8A**)]. These results were slightly modified when the G matrix was computed with the markers selected with a maf threshold of 5%: the relationship between number of offspring and inbreeding in *Q. petraea* became positive while remaining non-significant. Thus, whatever the significance and sign of the relationship, inbreeding depression signals were found to be very weak for both traits, within both species (**Figure 8**). Finally, when G matrix is computed over all the individuals without subdividing by species, inbreeding had a significant negative effect on both growth (coefficient = −107.41, *P*-value = 0.02) and reproductive success (coefficient = −1.94, *P*-value = 0.02).

## Discussion

### Targeted Sequence Capture Is a Reliable, Reproducible, and Transferable Marker Technique for Population Genetics Studies in Oaks and Beyond

Using targeted sequence capture, we successfully sequenced a large number of target genomic regions in a single assay. We obtained robust and reproductible target-enrichment results over several hundred samples, despite the use of only one *Q. robur* individual to design the capture probes. We evaluated the performance of target enrichment according to several parameters (number of captured targets, number of reads on target, length of targeted sequences, and sequencing depth). Two of these parameters varied considerably between experiments, providing a cause of concern, at first sight, for SNP detection. First, as observed in other studies (Albert et al., 2007; Fu et al., 2010) about 25% of the sequencing reads mapped to the targeted regions. A low proportion of the reads would be expected to be on-target for complex genomes, such as those of plants, which consist largely of repetitive sequences and TEs (52% of the *Q. robur* genome), making it difficult to design highly specific capture probes. The duplicated nature of many of the genes in most plants, particularly in trees (Plomion et al., 2018), adds another layer of difficulty in terms of specificity. In this study, the length of sequencing reads was positively correlated with the percentage of on-target sequences. As the length of sequencing reads was increased to 190 bp, the proportion of reads correctly aligned with their targets increased significantly (Supplementary File 4). This may reflect the relatively large size of the Agilent probes (120 bp) and the requirement of a sufficiently long target sequence fragment for correct hybridization. Despite the small size of the on-target fraction, it was sufficiently large to cover most of the target sequences deeply enough for the detection of a very large number of polymorphic sites in specific areas of the oak genome. Second, we observed significant variation in the number of sequences generated per sequencing run. The number of reads generated differed by a factor of up to two, but the small number of samples pooled per run (15) guaranteed that sufficient reads were produced to cover most of the target sequences with a sufficient depth in all samples. However, alternative NGS sequencing platforms, such as the Illumina NextSeq sequencing system (Illumina©), which is able to provide up to 400 million reads per run, should be considered, as such systems would make it possible to multiplex a larger number of samples per run, thereby decreasing the cost per sample analyzed.

Despite the variation of coverage and of the number of reads on target, this approach made it possible to recover SNPs with sufficient reliability, not only in genic regions, but also in intergenic regions. Indeed, even in intergenic regions, which are known to be highly redundant in plants, we managed to obtain a sufficiently high sequencing depth to detect a large number of SNP markers. Intergenic markers are particularly important for population genetic studies, in which they may be considered as neutral regions of the genome for the formulation of hypotheses relating to genetic diversity. They may also make a significant contribution to phenotypic variation. Li et al. (2012) showed that intergenic regions in maize play a significant role in quantitative trait variation, particularly for the 5 kb window upstream of the gene. These areas are enriched in trait-associated SNPs. There are, therefore, several complementary reasons for which intergenic regions should also be explored in population and quantitative genetic studies. Our study provides a large resource of genic and intergenic SNPs for the exploration of polymorphisms of QTLs previously identified as involved in the response to root waterlogging (Parelle et al., 2007) and bud phenology (Derory et al., 2010). Using target sequence enrichment, we targeted not only candidate genes previously identified as involved in drought resistance, response to waterlogging and bud phenology, but also sequences displaying differentiation within species or populations, of displaying significant genotype–phenotype or genotype–environment associations. Such large numbers of polymorphisms in these genomic areas would never have been identified with other methods, such as RADseq. Unlike capture data, RADseq data display a high variability of sequencing depth across loci, thus limiting the detection of polymorphic sites to genomes with a high level of coverage (Harvey et al., 2016). However, even in areas with high coverage, RADseq data have been shown to include a much higher proportion of singleton alleles, consistent with a high proportion of spurious allele calls (Harvey et al., 2016).

We studied the reproducibility of DNA capture by including three replicates in our design. We performed independent DNA extractions from the same tissues, constructed independent libraries and sequenced three replicates in different sequencing runs. Our assay was highly reproducible between replicates, as we obtained very similar results for each metric. We also compared the sets of SNPs independently detected in each replicated individual. The variability of sequencing depth between runs explains the identification of non-identical sets of SNPs (60–80%). However, 99.9% of the SNPs identified in each replicated individual were identical. For the retrieval of all polymorphic sites, we would need to increase sequencing effort (i.e., coverage) at the targeted loci. Finally, we also obtained reproducible results (in 97.67% of the cases) with another genotyping assay (mass spectrometry), for a set of 250 trees genotyped for 25 SNPs with both technologies (Truffaut et al., 2017).

One of the key assets of sequence capture technology is its ability to capture orthologous loci in closely related species. Capture efficiency and coverage decrease with increasing divergence between species. However, it is reasonable to think that a subset of design probes remain useful at the genus/family level, particularly for slowly evolving genes. The efficiency of sequence capture between species has been studied with animals. George et al. (2011) used a target capture method designed for humans on four monkey species. Despite sequence divergence of up to 4% between humans and monkeys, they were able to capture 96% of the target sequences. We used targeted probes designed for the *Q. robur* genome (Plomion et al., 2018) to recover the corresponding target sequence in three other species from the Fagaceae: *Q. petraea*, *Q. suber*, and *F. sylvatica*. Even if *Q. petraea* and *Q. robur* are considered to be separate species, they belong to the same species complex and can hybridize (Lepais et al., 2009). It has recently been shown that *Q. petraea* and *Q. robur* share a mosaic of genes that have crossed species boundaries (Leroy et al., 2017). Logically, as divergence between these two species is very limited, we expected the detection of orthologous sequences in *Q. petraea* to be straightforward. We also considered a more distantly related species from the same genus (*Q. suber*) and another species (*F. sylvatica*) from a different genus belonging to the same botanical family (Fagaceae). No whole-genome sequence is yet available for *Q. suber* or *F. sylvatica*. However, transcriptomic assemblies are available for both species (Pereira-Leal et al., 2014; Lesur et al., 2015). These genetic resources limit the possibility of identifying markers outside the exonic gene space. Without a complete reference genome, it is not possible to detect many of the polymorphisms in intronic and intergenic regions. We were able to capture sequences from both species, despite the use of much smaller numbers of cork oak and beech trees (2) than of *Q. robur/Q. petraea* trees (293). It would, therefore, be reasonable to expect the detection of a much larger number of markers if a larger number of genotypes were considered. As expected, we clearly showed that, with increasing divergence time, the number of captured target sequences and the fraction of their length captured decrease. The number of SNPs detected in *Q. suber* and *F. sylvatica* also decreased with decreasing sequencing depth. Given that the probes were designed based on the *Q. robur* reference genome (Plomion et al., 2018), probe hybridization was less efficient for cork oak and beech samples, resulting in partial hybridization, lower levels of coverage, and the capture of shorter sequences. Nevertheless, we were still able to detect several thousand SNPs in each species.

Our findings demonstrate that this sequence capture assay for the targeted resequencing of oak genomic regions is a cost-effective strategy for generating orthologous markers in related species of the Fagaceae family in the absence of a reference genome.

### Estimation of Relatedness Among Individuals in a Wild Oak Population

We aimed to develop SNP markers for assessing genetic relatedness in natural populations, with a view to estimating genetic parameters and breeding values in evolutionary studies. Genetic relatedness has traditionally been assessed by determining pedigree relationships over multiple generations. However, this approach is difficult to implement for long-lived species, such as oaks, due to obvious biological and logistic constraints. We therefore attempted to estimate genomic relatedness among trees within a single generation, to find ways of conducting quantitative evolutionary studies in the wild. The genomic relatedness estimated with a large number of molecular markers has already been shown to be more efficient than pedigree relationships for the purposes of prediction (Kardos et al., 2015). We show here that genomic capture is a promising molecular technique for this purpose.

Our targeted sequence capture approach generated thousands of genotype-specific single-nucleotide variants, making it possible to determine the relatedness between individuals with a high degree of precision. The precision of relatedness estimates increased only slightly when the number of markers increased above a few thousand.

The lack of variation of realized relatedness with the number of markers used (i.e., between maf = 1% and maf = 40%), beyond a few thousand markers, suggests that several thousand markers are sufficient for the estimation of relatedness between individuals and, thus, of the level of inbreeding of each individual (relative to the population). By contrast, we found that the use of a much more limited number of markers (a few tens), as in most traditional population genetic investigations, results in a broad scattering of genomic relatedness values around the expected value, with a tendency toward a large sampling variance. However, our results also show that the genomic relatedness estimated with thousands of markers is systematically slightly lower than that predicted on the basis of pedigree, at least for oaks. A similar trend has been reported for other forest trees (e.g., Bartholomé et al., 2016) and this pattern is predictable, given that allelic frequencies are estimated from data for a population of related individuals (Wang and Zhang, 2014; Kardos et al., 2015). Indeed, simulation studies showed that the difference in the proportion of identity by descent (IBD) between the genomes of individuals within populations is systematically overestimated when IBD is estimated on the basis of the level of homozygosity expected at population level under neutral conditions (Kardos et al., 2015). In this case, we expect a systematic downward bias in the estimation of relatedness.

### Within-Population Inbreeding Depression Is Weak, but Differs Between Closely Related White Oak Species

Inbreeding depression can be defined as a decrease in fitness trait (i.e., survival, fertility, or growth) values in the most inbred individuals (Charlesworth and Willis, 2009). Studies of inbreeding depression in natural populations were long characterized by the difficulty of accurately estimating inbreeding coefficients. In traditional population genetic studies, using a few tens or hundreds of markers, heterozygosity is often used as a proxy for inbreeding and fitness values are regressed against heterozygosity level to estimate inbreeding depression. This method has been shown to be imprecise, partly because heterozygosity at a few loci is not necessarily correlated with inbreeding (Szulkin et al., 2010 and references therein). With the thousands of markers developed in this study, it should be possible to estimate relative inbreeding levels more precisely between individuals.

The method we used to estimate the genomic relatedness matrix provides values for inbreeding (and relatedness) relative to population allelic frequencies. Thus, inbreeding values cannot be used to compare homozygosity levels between individuals from different species. However, when estimating inbreeding by considering all individuals (i.e., pure *Q. petraea*, pure *Q. robur*, and admixed genotypes) to belong to the same population, individuals assigned to the species *Q. robur* tend to be more inbred overall than individuals assigned to the species *Q. petraea*, and, as expected, individuals classified as admixed are less inbred than “pure” individuals (data not shown).

Our results showed that, within species, growth was not affected by inbreeding depression in either species. However, reproductive success within species was characterized by weak inbreeding depression in *Q. petraea*, whereas no such trend was observed in *Q. robur*. A comparison between species showed that both growth and reproductive success were significantly lower in *Q. robur* than in *Q. petraea*. Thus, even though no particular pattern was detected within species, *Q. robur* individuals tended to be more inbred than *Q. petraea*, suggesting that *Q. robur* is more affected by inbreeding depression than *Q. petraea*.

As illustrated in a recent paper (Truffaut et al., 2017), the two species occupy contiguous areas in the study plot (i.e., *Q. petraea* trees are in the north-east and *Q. robur* trees are in the south-west). Thus, as individuals compete principally with the surrounding trees, competition on this plot can be considered to occur principally within species. Given that both these species are outcrossers and form large populations (Gerber et al., 2014), strong inbreeding depression would be expected, due to an accumulation of deleterious alleles (genetic load). However, our results in this study do not support this hypothesis. There are two non-exclusive interpretations for these observations. First, inbreeding levels tend to be extremely low in adult trees (the trees were roughly 100 years old), probably because the individuals with the highest levels of inbreeding are eliminated, by natural or human-mediated selection, when the stand is young. Second, a context of strong selection and competition may have reduced the inbreeding load (Hedrick et al., 1999; Whitlock, 2002; Agrawal, 2010), by purging deleterious mutations. However, the negative correlation between fitness and inbreeding level observed when considering all individuals (from both species) may reflect the spatial distribution of the species in this mixed forest plot, resulting in weaker competition between individuals from different species, attenuating “between species” selection. As both species display differences in fitness-related traits correlated with differences in inbreeding coefficient, *Q. petraea* seedlings might be expected to outcompete *Q. robur* in mixed stands. Interestingly, these expectations are supported by recent observations showing that *Q. petraea* seedlings have gained ground over *Q. robur* seedlings in one generation (Truffaut et al., 2017).

Finally, inbreeding may also be limited by an even-age silvicultural regime. In such systems, in which all trees within a given plot belong to the same age class, mating between relatives, and between parents and offspring in particular, is avoided, which is not the case in other systems involving trees of different ages (Finkeldey and Ziehe, 2004).

## Conclusion

We demonstrate here that the combination of targeted sequence capture with next-generation sequencing is an efficient method for studying genetic diversity, at genome scale, in natural oak populations. We show here that this method is highly reproducible and can be extended to related species within the Fagaceae. We also used this technique to assess realized genomic relatedness in natural oak populations. We found that this method could be used to retrieve relationships predicted by pedigree relatedness. Given our results and those of previous SNP transferability studies (Lepoittevin et al., 2015), we conclude that this method can be applied to a large number of white oak species, but also to other more distant oak species from other botanical sections (Hubert et al., 2014) or related genera of the Fagaceae family. We used this method to assess genetic relatedness and inbreeding coefficients, but it could also be used for other purposes requiring a very large number of markers, such as phylogenomics, phenotype–genotype–environment associations, and the prediction of breeding values for relevant traits. This work paves the way for evolutionary and genetic studies *in natura* in long-lived tree species, such as oaks, that are difficult to study in controlled or common garden conditions.

## Data Availability

Sequencing data for the 300 samples considered in this study are available in the NCBI – SRA database under the BioProject *PRJNA445867*. The haploid version (scaffolds) of the *Q. robur* genome (haplome V2.3) has been deposited on the EMBL – ENA database under accession *OLKR01000000.* The set of 191,281 polymorphic sites between *Q. petraea/Q. robur*, *Q. suber*, and *F. sylvatica* associated with each trait detailed in **Table 1** is available through the EVOLTREE eLab service: available from: http://www.evoltree.eu/index.php/snp-db. The list of candidate genes included in the set of target sequences, the 33,931 probe sequences, the description of the probes along with their transferability, and the analysis scripts used in our study can be found on the TreePeace website under the *Publications* tab: available from: http://www.treepeace.fr/?page_id=1401.

## Author Contributions

IL contributed to the conception of the work, performed SNP detection, evaluated the success of the sequence capture experiment, and contributed to the writing of the draft manuscript. HA analyzed the genomic relatedness between individuals and contributed to the writing of the draft manuscript. CB was responsible for library construction and sequencing. EC was involved in sampling. CP supervised the sequence capture experiment and revised the manuscript. AK contributed to the conception of the work and revised the manuscript.

## Conflict of Interest Statement

The authors declare that the research was conducted in the absence of any commercial or financial relationships that could be construed as a potential conflict of interest.
