# Supplementary figures and images for: Development of Target Sequence Capture and Estimation of Genomic Relatedness in a Mixed Oak Stand
Source: Front Plant Sci. 2018 Jul 13;9:996. doi: 10.3389/fpls.2018.00996 (PMC6053538; doi:10.3389/fpls.2018.00996)

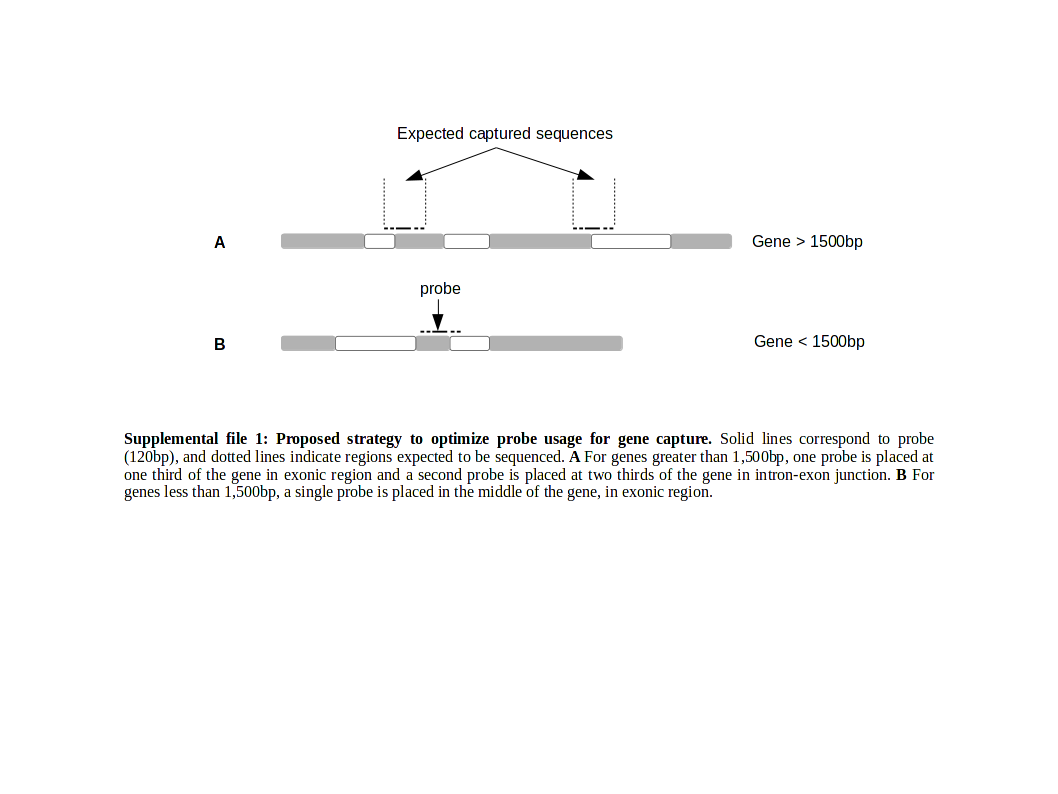

Supplement: Supplementary file 1 [file Image_1.TIFF]

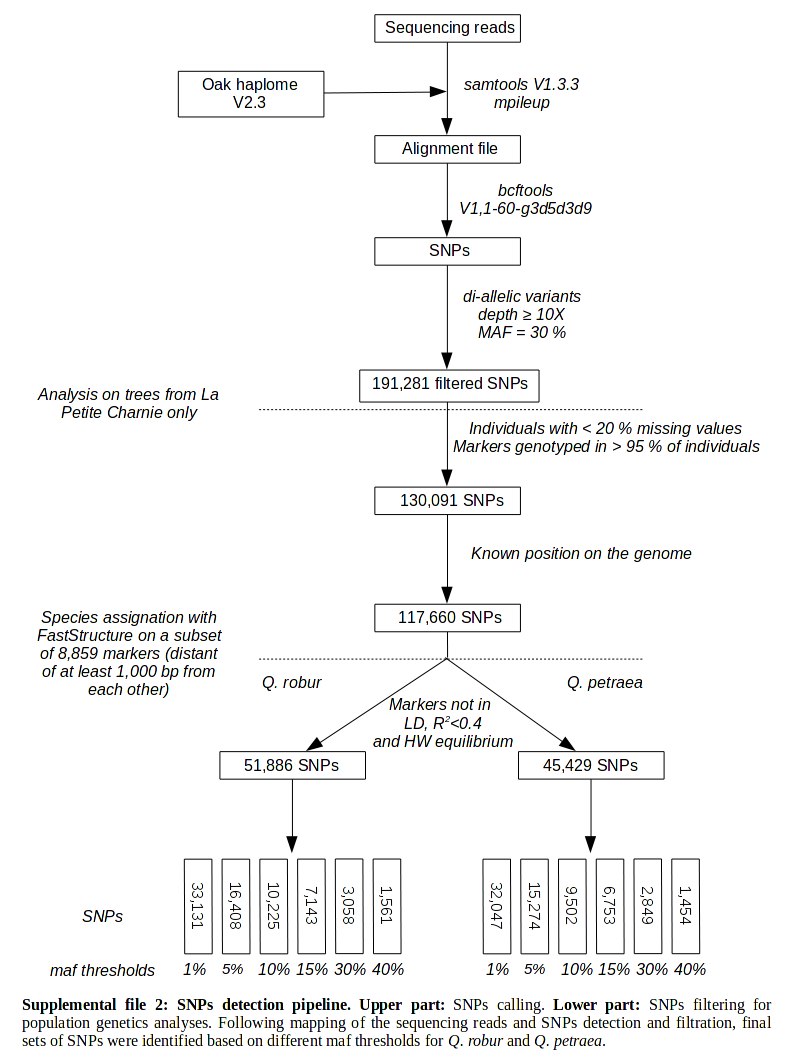

Supplement: Supplementary file 2 [file Image_2.TIFF]

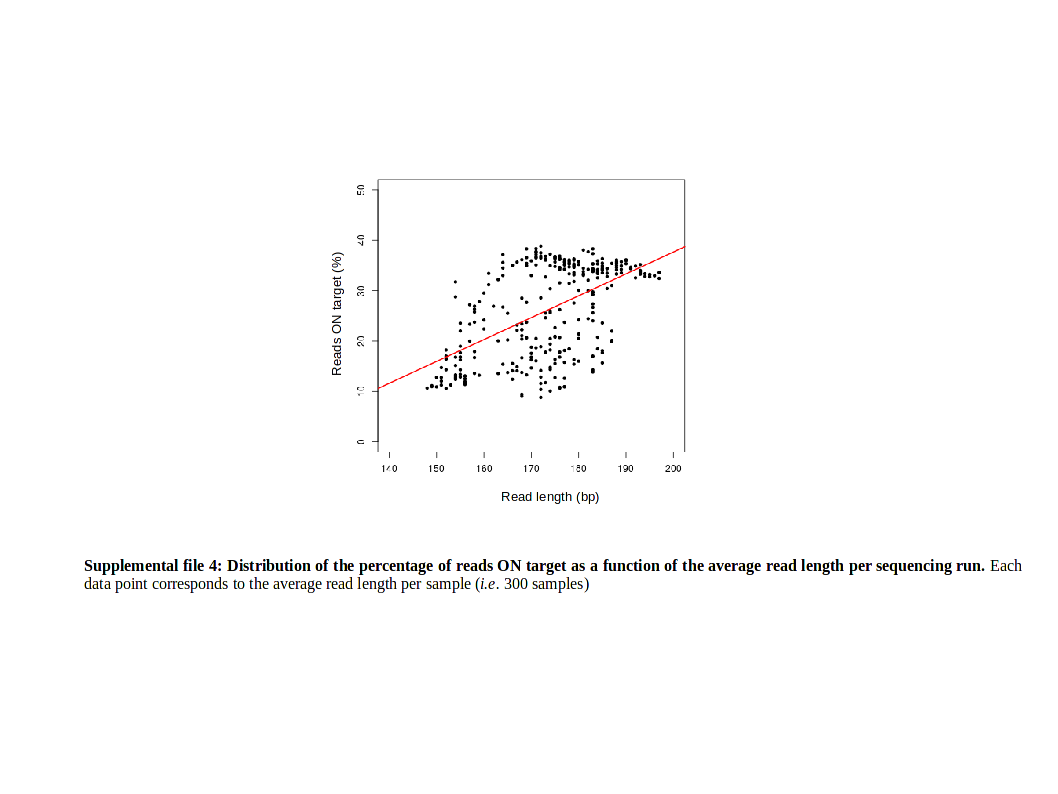

Supplement: Supplementary file 3 [file Image_3.TIFF]

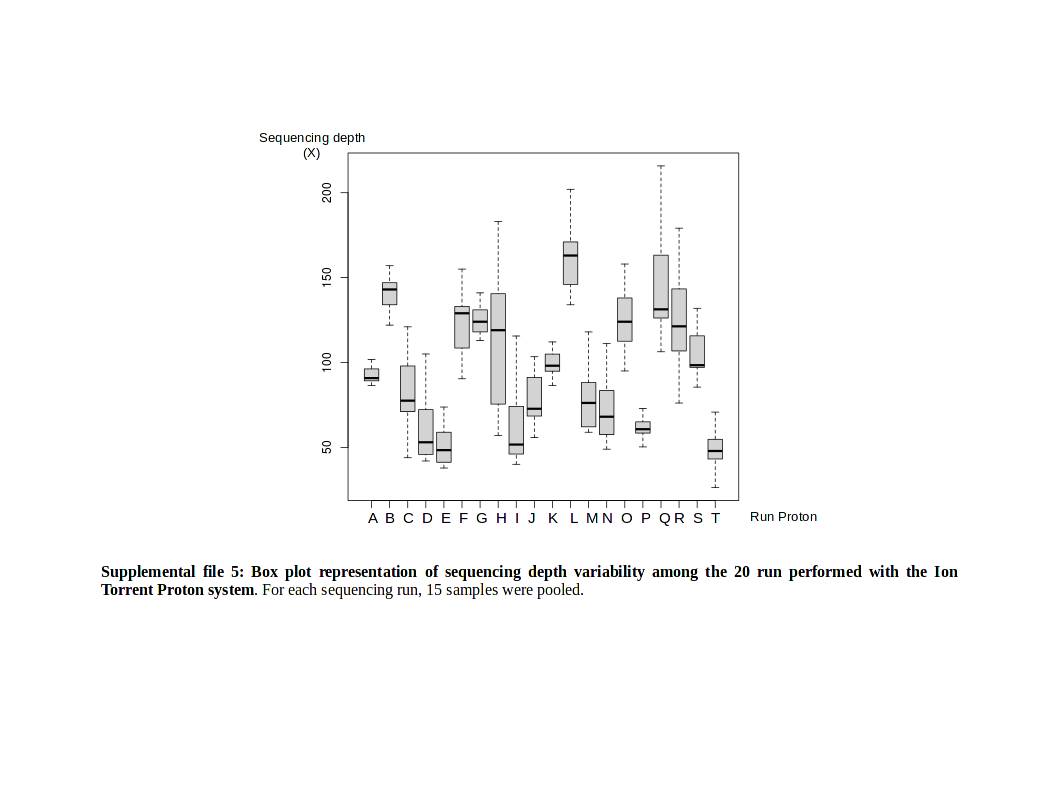

Supplement: Supplementary file 4 [file Image_4.TIFF]

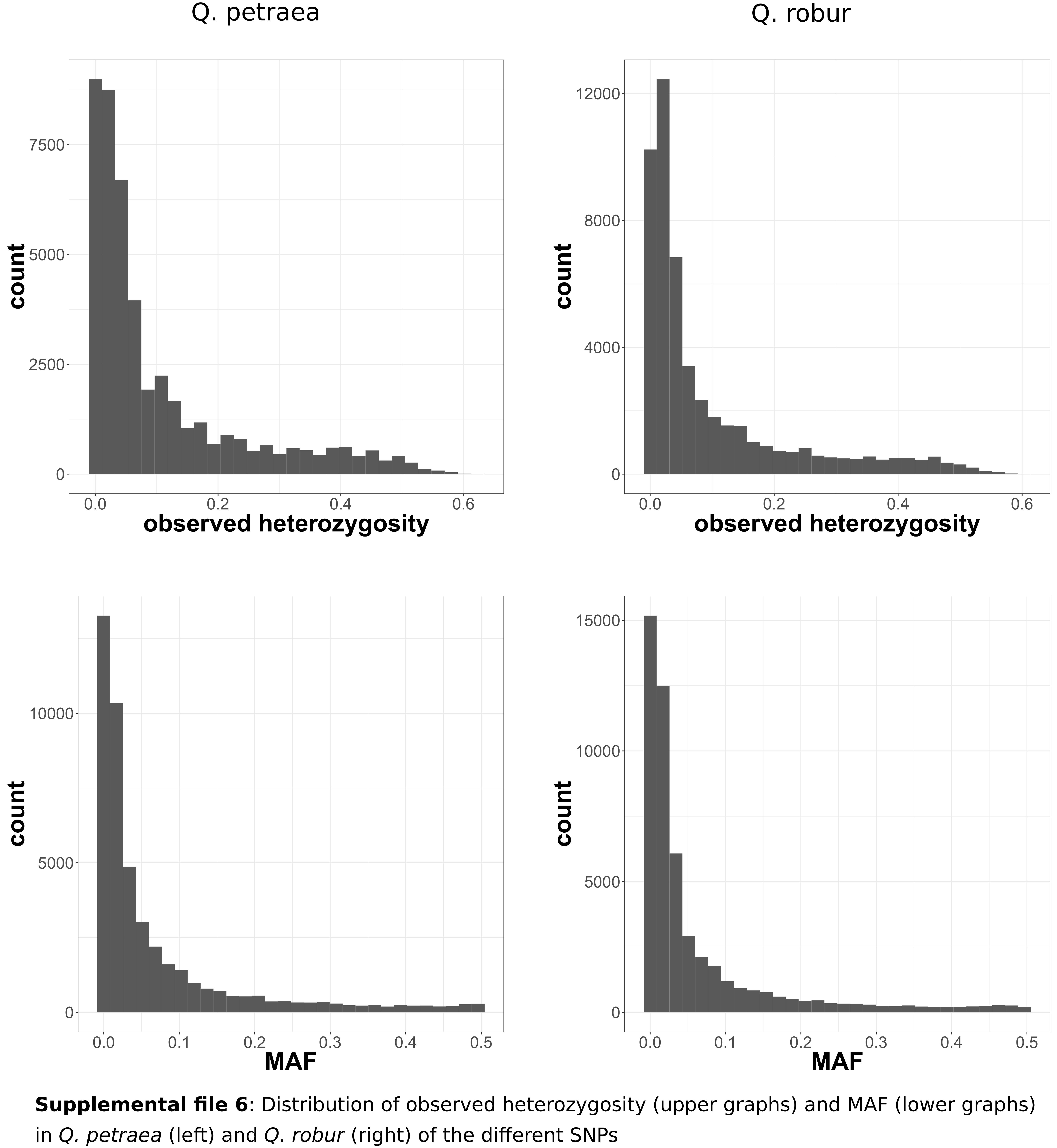

Supplement: Supplementary file 5 [file Image_5.TIFF]

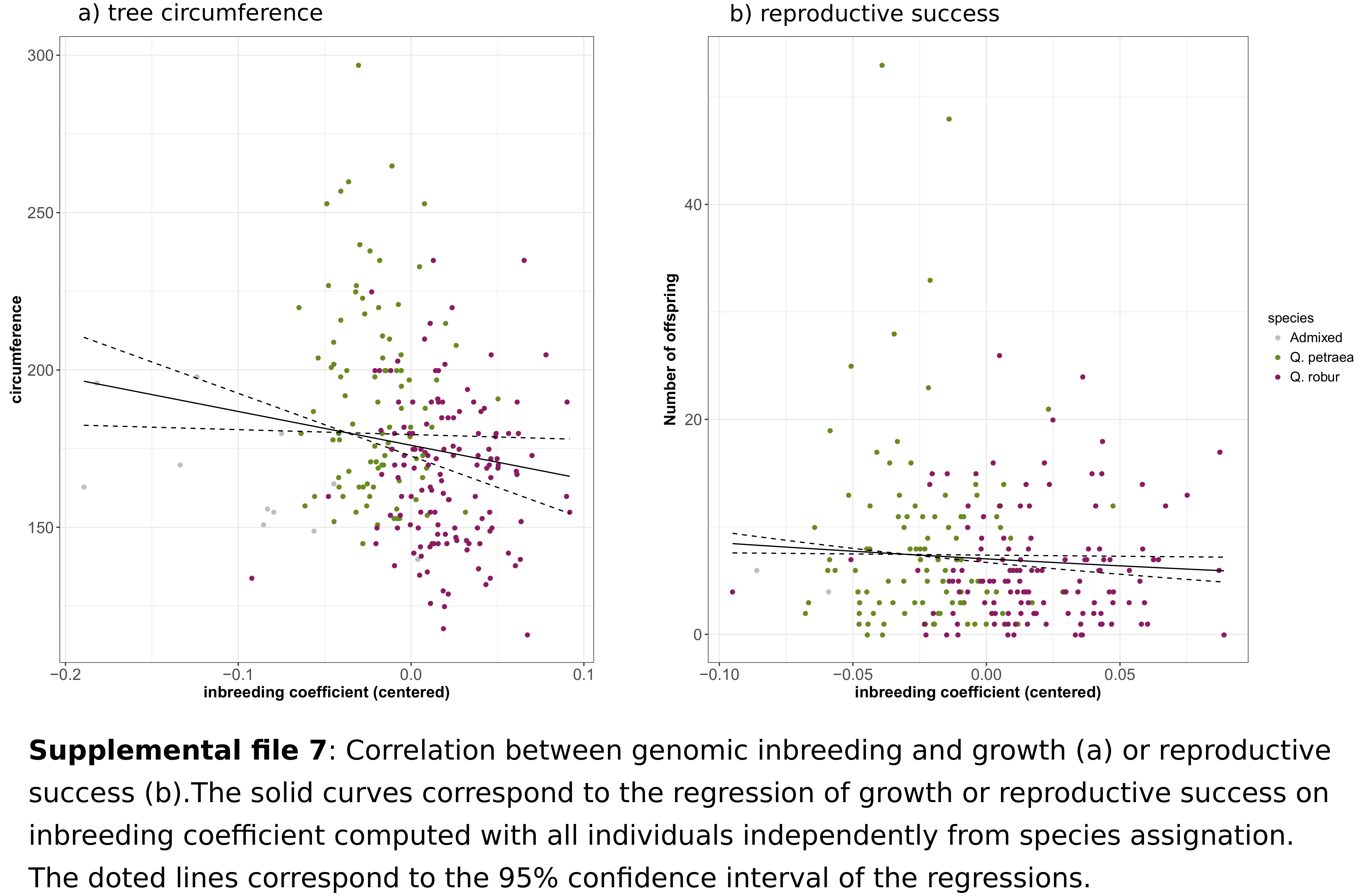

Supplement: Supplementary file 6 [file Image_6.TIFF]

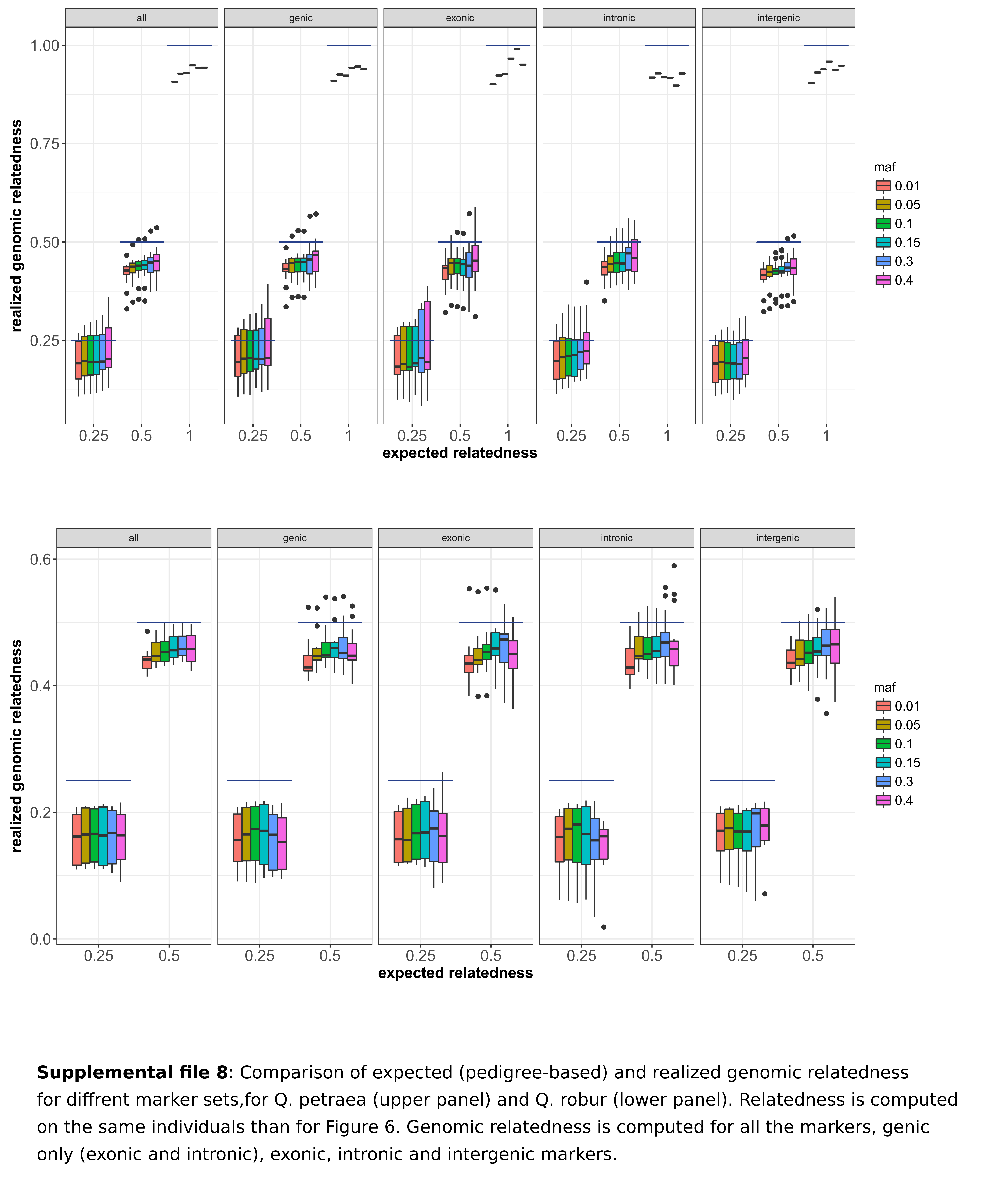

Supplement: Supplementary file 7 [file Image_7.TIFF]
