# Supplementary material for: Development of Target Sequence Capture and Estimation of Genomic Relatedness in a Mixed Oak Stand
Source: Front Plant Sci. 2018 Jul 13;9:996. doi: 10.3389/fpls.2018.00996 (PMC6053538; doi:10.3389/fpls.2018.00996)
Supplement: Supplementary file 8 [file Table_1.PDF]

Proton run	Number of reads	average read length	reads ON target	reads ON target(%)	Average sequencing depth
A	65,426,948	176.8	20,147,545	30.80	91.42
B	91,247,539	175.7	30,980,059	33.95	140.93
C	82,774,044	158.9	20,144,514	24.34	77.32
D	85,708,344	156.6	15,724,373	18.35	64.32
E	85,535,163	156.2	12,545,489	14.67	50.51
F	88,301,912	170.3	27,849,048	31.54	113.24
G	84,347,068	170.3	27,604,493	32.73	123.67
H	96,937,444	178.1	22,422,662	23.13	113.73
I	101,853,924	162.4	23,921,269	23.49	64.87
J	80,219,832	179.7	16,829,370	20.98	78.73
K	66,170,197	180.5	21,860,451	33.04	100.07
L	95,625,571	186.3	33,238,344	34.76	161.67
M	92,446,036	168.1	17,093,687	18.49	78.77
N	84,118,474	172.9	16,319,193	19.40	73.14
O	134,977,869	175.8	25,730,817	19.06	120.60
P	76,522,560	183.2	12,916,956	16.88	62.17
Q	88,516,362	194	28,324,777	31.99	142.02
R	83,045,232	187.9	26,393,803	31.78	124.30
S	71,112,637	171.2	23,258,910	32.71	106.25
T	90,349,277	174.9	10,780,269	11.93	48.39
**20 runs**	**87,261,821**	**174**	**21,704,301**	**25.20**	**96.81**

Supplemental file 3: Statistics of the 20 Proton sequencing runs. For each run, the number of reads produced, the average length of the reads, the number and the percentage of reads ON target, the average sequencing depth are listed.
